# A Study of Pandemic Prevention Health Behavior in Adults

**DOI:** 10.3390/ijerph19138181

**Published:** 2022-07-04

**Authors:** Mihyeon Seong, Kyungeui Bae

**Affiliations:** 1 Department of Nursing, Changshin University, Changwon 51352, Korea; mihyeon0624@cs.ac.kr; 2 Department of Nursing, Dongseo University, Busan 47011, Korea

**Keywords:** COVID-19, pandemic prevention, health belief model, perceived behavioral control, perceived disability, perceived severity, subjective norms, TPB

## Abstract

Following the outbreak of COVID-19, the World Health Organization recommended prevention measures to minimize the spread of the pandemic. However, strict compliance with prevention measures requires positive health behavior practices, especially among adults. Therefore, this study investigated adults’ health behaviors in relation to pandemic prevention based on the health belief model (HBM) and the theory of planned behavior (TPB). This study used a structural model, applying the HBM and the TPB to explain and predict pandemic prevention behaviors in adults. The data obtained were statistically analyzed using SPSS 25.0 and AMOS 25.0. The results revealed that, in total, 341 adults (age: 20–64 years; males: 167, females: 174; single: 167; married: 164; divorced: 8) participated in this study. Of the 341 participants, 339 had use of the internet and a smartphone. Furthermore, the results revealed that attitudes, subjective norms, and perceived behavioral control in relation to pandemic prevention behaviors directly affected people’s intentions to adopt pandemic prevention behaviors. Perceived severity and perceived disability had significant indirect effects on the intention to prevent pandemics; pandemic prevention behavior and perceived behavioral control had a significant effect on pandemic prevention intention. The pandemic prevention education programs suggested in this study have the potential to improve adults’ health behavior in relation to pandemic prevention.

## 1. Introduction

Following the outbreak of the novel coronavirus SARS-CoV-2 (COVID-19) in Hubei Province, China, in 2019, the WHO Emergency Committee declared an international emergency under the International Health Regulations (IHR) on 30 January 2020 [1]. With its rapid transmission rate, COVID-19 has significantly changed people’s lives and affected many aspects of the global, public, and private economies [2]. Border closures and economic downturns, as well as uncertainty and fear related to viral outbreaks, have already had a number of psychological effects on people [2,3].

As was the case with previous pandemics, such as the Ebola or Zika viruses, mass media and the internet have become mechanisms for spreading misinformation, which has influenced public health behavior and health-related decision making [4,5,6]. People are anxious to know what they can do to prevent and treat the disease. Currently, due to different opinions about the use of antiviral treatment for COVID-19, preventive action has become essential [6,7].

Each country has proposed different preventive measures in response to COVID-19, such as frequent hand washing, public health measures, including social distancing, and the postponement or cancellation of large gatherings [8]. Furthermore, the WHO stated that, to prevent the spread of COVID-19, coordination mechanisms should be developed that relate not just to personal health, but also to different sectors of society, such as transportation, travel, commerce, and finance [9]. As a result, various countries recommend avoiding crowded places, implementing good hand hygiene, the use of personal protective equipment, and social distancing in homes, hospitals, public places, and workplaces. Schools and workplaces have been closed where necessary. The number of infections is increasing exponentially in countries where such health practices are not compulsory [10], and there is a need to understand people’s health behaviors in relation to pandemics. This study aims to investigate pandemic prevention behavior through various theories of health behavior, such as the health belief model (HBM) and the theory of planned behavior (TPB).

## 2. Theoretical Background

HBM is a theory that applies behavioral science to health promotion and is the most widely used theory of health behaviors [11]. The HBM assesses the attempts of individuals to avoid or recover from disease, with the expectation that certain behaviors can prevent or alleviate diseases [12]. Meanwhile, the TPB is known to predict behavioral intentions through behavioral attitudes, subjective norms, and perceived behavioral control, which allows for a simple and accurate understanding of what people want to do [13]. The TPB proposes that the intention of action acts as a direct determinant of human action [14], and the attitude toward behavior refers to the positive or negative evaluation of an individual’s behavior. Subjective norms are social factors that individuals perceive as social pressures either to act or not to act. In addition, perceived behavioral control reflects past experiences or predicted obstacles in the future, which informs the individual’s perception of the ease or difficulty of performing the behavior [14]. Therefore, the TPB is reported to predict health behavior by measuring intentions such as drinking, smoking, and illegal drug use [13]. Many studies have used the theory to predict various behaviors, such as vaccination intention, smoking cessation behavior, drunk driving behavior, and health behavior [15,16,17,18]. The TPB is clearly specified by the measurement and calculation of hypothesized causal relationships between constituent variables, providing a conceptual framework for identifying and understanding the causes or beliefs that motivate preventive actions [12]. Therefore, these two health-behavior-related theories are very helpful in identifying and understanding factors that influence public health behavior [19], and applying these theories can reveal the direct determinants of public beliefs, behavioral controls, intentions, and behavior, which is useful for explaining and predicting behavior [20,21].

Before individuals undertake particular actions, they are influenced by personal factors, such as their skills, abilities, and knowledge related to the given behavior, and some external factors, such as time, opportunity, and the cooperation of others [22]. The intentions, attitudes, and beliefs that determine intentions play an important role in accurately predicting a specific behavior, and behaviors are initiated and continued based on these antecedent factors [23]. Furthermore, the attitudes of individuals are formed by antecedent factors, such as the general characteristics of the individual, and this information is a factor that explains individual beliefs [23]. Therefore, this information is useful for formulating intervention strategies for preventing new pandemics.

While HBM- and TPB-based studies of intentions towards pandemic prevention in the adult general population are scarce, these two models have been applied to the following subjects: nurses’ prevention actions against needle stick injury [21], health behavior and self-medication in patients with pulmonary tuberculosis [19], and the development of a nutrition education program for reducing sodium intake [24]. However, there are few studies related to COVID-19 or the pandemic and, as COVID-19 continues to have long-lasting effects worldwide, all members of society are exposed to the risk of infection [25]. Therefore, this study intends to apply the health belief model and the planned behavior theory to identify the factors affecting the pandemic, one of the major health problems and issues facing society, and to predict the behaviors that can help to prevent it. This is expected to provide basic insight into people’s health behaviors in a pandemic situation, and will make it possible, when another pandemic comes, it is predicted that it will be possible to improve the prevention behavior and the improvement of performance rate by being able to determine the priority of preventive behavior and education that can improve the knowledge and attitude related to preventive behavior [26].

Therefore, this study intends to apply the HBM and the TPB to identify the factors that affect attitudes toward pandemic prevention. 

### 2.1. Purpose

This study aimed to analyze factors related to pandemic prevention behaviors in adult; the analysis is based on the HBM by Becker [27] and the TPB by Ajzen [23], and is conducted with the possibility of applying the derived results to the actual field. Specifically, the study identifies: (1) the general characteristics of adults, the perceived severity of the health problem, adults’ perceived disabilities, attitudes toward pandemic prevention behavior, subjective norms, perceived behavioral control, and behavioral level; (2) the effects on pandemic prevention behaviors of attitudes, subjective norms, and adults’ perceived behavioral controls related to pandemic prevention behaviors; (3) whether the perceived severity and disability of adults affect their attitudes toward pandemic prevention behavior, subjective norms, and perceived behavioral control; (4) the effects of intentions related to pandemic prevention behavior and perceived behavioral control on pandemic prevention behavior.

### 2.2. The Conceptual Framework of the Study

This study selected the perceived severity of the problem and perceived disability in the HBM by Becker [25] to construct a predictive model for pandemic prevention in adults. A conceptual framework was constructed based on attitudes toward behavior, subjective norms, perceptions of behavioral control, implementation intention, and behavior, which are the main concepts of the TPB proposed by Ajzen [23]. In the TPB, attitudes toward behavior, subjective norms, perceived behavioral control, and intention have antecedent factors, such as behavioral beliefs, normative beliefs, and control beliefs. These beliefs are related to the HBM, in which behavior is predicted depending on the subjective perceptions of individuals [27,28]. Based on this, perceived severity and perceived disability have been selected as variables. Therefore, the conceptual framework of this study is that the perceived severity and perceived disability of HBM affect behavioral attitudes, subjective norms, and perceived behavioral control according to TPB. Attitudes toward behavior, subjective norms, and perceived behavioral control affect performance intentions. Performance intention affects behavior, and perceived behavioral control constitutes a framework that directly affects behavior. 

### 2.3. Research Hypothesis

Based on previous studies, the hypotheses derived by applying the health belief model and the theory of planned behavior are as follows [19,21,24].

**Hypothesis** **1.** *Adults’ health beliefs about pandemic prevention behaviors will have a significant direct effect on their attitudes toward pandemic prevention behaviors, subjective norms, and perceived behavioral control*.

**Hypothesis** **2.** *There will be a significant correlation between pandemic prevention attitudes, subjective norms, and perceived behavioral control in adults*.

**Hypothesis** **3.** *Adults’ attitudes toward pandemics will not only have a significant direct effect on their pandemic prevention intentions but will also have a significant indirect effect on their prevention behaviors, by increasing their intention to prevent pandemics*.

**Hypothesis** **4.** *The subjective norms for pandemics in adults will not only have a significant direct effect on the intention to prevent a pandemic but will also have a significant indirect effect on the prevention of pandemics by increasing their intention to prevent a pandemic*.

**Hypothesis** **5.** *The perceived behavioral control for a pandemic in adults will not only have a significant direct effect on their pandemic prevention intentions, but will also have a significant indirect effect on their pandemic prevention behavior by increasing their pandemic prevention intentions*.

**Hypothesis** **6.** *The pandemic prevention intentions in adults will have a significant direct effect on their pandemic prevention behavior*.

**Hypothesis** **7.** *The perceived behavioral control for pandemics in adults will have a significant direct effect on their pandemic prevention behavior*.

## 3. Materials and Methods

### 3.1. Research Design and Hypothetical Models

**Figure 1:**
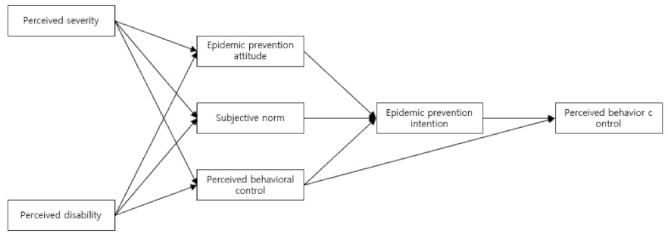
Modified the HBM and the TPB Model for the research.

This study used a structural model by testing the fit of model data and research hypotheses after presenting a hypothetical model for the prevention of pandemics in adults. The HBM and the TPB are partially applied to this model to explain and predict pandemic prevention behaviors in adults. See Figure 1.

### 3.2. Participants

In order to reduce the influence of exogenous variables on the research results, the subjects of this study were randomly sampled and, after IRB approval, a survey was conducted using an internet portal site or a specialized research institute using a Google form. Adults aged 20 to 64 years were targeted. Data collection by the questionnaire can be affected by exogenous variables, such as the situation at the time of response or the respondent’s mood; nevertheless, this method was selected because using a standardized questionnaire can increase the comparability of the results [29]. In order to protect the respondents identities and personal information, we did not collect personally identifiable information. In addition, the subjects selected for this study were those who understood the purpose of the study and voluntarily agreed to participate in it, and those who could participate in the survey using a smartphone or the internet. It was recommended that the number of participants required for the structural equation model should be 15 per measurement variable [30]. Furthermore, based on the opinion that there should be 200 or more participants when using the maximum likelihood method [30], it was preferable to recruit between 200 and 400 participants [31]. The recruitment target was set at 300 people, and data were collected from 360 people, taking into consideration the 20% dropout rate. Finally, the valid responses of 341 participants were analyzed, while the invalid responses were excluded.

### 3.3. Measures and Scales

#### 3.3.1. Health Beliefs Related to Pandemic Prevention Behavior

In this study, the scale developed by Choi et al. [32], which was modified and supplemented by Lee and Park [33], was used to measure the perceived sensitivity, perceived severity, perceived benefit, and perceived disability of the pandemic prevention behavior, after additional modification and supplementation by the authors to meet the purposes of this study. The questionnaire measured beliefs using self-reporting, and bias in the responses has the potential to threaten the validity of the questionnaire. In particular, there is a tendency to base responses to questions about individual beliefs on social desirability or impression management, which are socially-constructed behaviors, rather than on one’s original beliefs. Inverse questions can be produced to reduce the effect of such biases, but recent studies argue that inverse questions are not a good way to deal with response bias [34,35]. Therefore, this research was revised and supplemented to suit the purposes of the study. In addition, the validity of the response structure was analyzed through exploratory factor analysis. Each item was scored on a five-point Likert scale from 1 to 5 points. In the study by Lee and Park [33], Cronbach’s α was 0.74 for perceived severity and 0.62 for perceived disability. In this study, Cronbach’s α was 0.66 for perceived severity and 0.66 for perceived disability.

#### 3.3.2. Attitude toward Pandemic Prevention Behavior

The scale developed by Seong et al. [36] was used to measure pandemic prevention attitudes. The Epidemic Attitude Tool was developed to measure attitudes that reflect the characteristics of a pandemic, such as ‘I think we should pay more attention to personal hygiene when there is an epidemic’. The pandemic prevention attitude scale, consisting of a total of 20 items, was developed to measure attitudes that reflect the characteristics of a pandemic. The scale consisted of eight items related to personal belief attitudes, five items related to knowledge attitudes, two items related to normative attitudes, three items related to affective attitudes, and two questions related to intentional attitudes. These items were scored on a 5-point Likert scale, with 5 points for strongly agree, 4 points for agree, 3 points for neutral, 2 points for disagree, and 1 point for strongly disagree, with a higher score indicating a more advanced pandemic prevention attitude. In the study by Seong et al. [36], Cronbach’s α was 0.92, and, in this study, Cronbach’s α was 0.92.

#### 3.3.3. Subjective Norm

In this study, the subjective norm was defined as the pressure that the participants felt from the people around them to always comply with the pandemic preventive guidelines [37], It was measured according to two items: ‘I want it (washing hands, wearing a mask, keeping distance, etc.)’, and ‘I try to follow the opinions of people who are important to me’. The scale developed by Jeong and Kim [38] was used after modification and supplementation to meet the requirements of this study. As for the reliability of the scale, Cronbach’s α was 0.87 in the study by Jeong and Kim [38], and Cronbach’s α was 0.77 in this study.

#### 3.3.4. Perceived Behavioral Control

Perceived behavioral control was defined as the difficulty that the subject felt about always following the guidelines for preventive activities during epidemics [37], i.e., ‘When I am very busy with work, after minor contact with people’. It consists of 3 items, such as ‘Do not follow social distancing’, etc. The scale developed by Jeong and Kim [38], which uses six items related to perceived control, a sub-domain of the hand hygiene behavior scale [31], was modified and supplemented according to the requirements of this study. As for the reliability of the scale, Cronbach’s α was 0.87 in the study conducted by Jeong and Kim [38], and Cronbach’s α was 0.91 in this study.

#### 3.3.5. Pandemic Prevention Intention

The pandemic prevention intention in this study referred to the intention of the participants to comply with hand hygiene. Additionally, five items on intention, a sub-domain of the hand hygiene behavior scale [39], were used, i.e., ‘I try to comply with the epidemic prevention guidelines when an epidemic is circulating’. The score of each question ranged from strongly disagree (1 point) to strongly agree (7 points), with a higher score indicating a higher level of hand hygiene behavior intention. Cronbach’s α was 0.74 for the scale at the time of development, and 0.86 in the study by Jeong and Kim [38].

#### 3.3.6. Pandemic Prevention Behavior

In this study, pandemic prevention behavior was measured by the COVID-19 infection prevention behavior scale developed by Kim Seon-joo et al. [40], which consisted of a total of 18 items, scored on a four-point Likert scale. The sub-factors of the scale consisted of distancing, quarantine rules, personal hygiene rules, and high-risk rules, scored as 4 points for “always,” 3 points for “often,” 2 points for “sometimes,” and 1 point for “never,” with a higher score indicating a higher level of COVID-19 infection prevention behavior. After obtaining the author’s permission, the tool was renamed through exploratory factor analysis, and items such as ‘If you think your hands are contaminated, wash your hands with water and soap or hand sanitizer’ were used with modifications and supplementations. In the study by Kim Seon-joo et al. [40], the Cronbach’s α was 0.90, but it was 0.85 in this study.

### 3.4. Data Collection and Ethical Considerations

The data for this study were collected from 2 March to 15 March 2022. Prior to data collection, approval was obtained from the Institutional Review Board (IRB) of C University in accordance with the declaration of Helsinki (approval number: CSIRB-R202147). This study was conducted by researchers from a specialized research firm who had been trained on the purposes and procedures of the research, as well as the ethical considerations. Prior to the survey, the purposes and procedures of the study, the contents of the study, and the possibility of stopping and withdrawing from the study were posted on the screen shown to the participants, to allow them to see all of this information. Data were collected using a self-reported questionnaire. At the time, due to the non-face-to-face nature of the investigation, obtaining written consent was impossible, thus the written consent was replaced by checking the consent items. The authors were responsible for managing the collected data; the survey data were stored on a hard disk with a designated password to prevent unauthorized access, and were processed on the personal computer of the authors. 

### 3.5. Data Analysis

The data of this study were statistically analyzed using SPSS 25.0 and AMOS 25.0, according to the following procedure:

First, exploratory factor analysis was performed to analyze the validity of the scale, and the reliability of the items constituting the factors was analyzed using Cronbach’s ⍺. 

Second, descriptive statistics analysis was performed to understand the general characteristics of the participants and the level of the variables. 

Third, Pearson’s correlation was used to examine the correlation between the variables.

Fourth, to verify the validity of the observed variables constituting the latent variables, confirmatory factor analysis was performed, and the convergent validity and discriminant validity were verified. 

Fifth, structural equation model analysis was conducted to verify the direct and indirect effects between variables. A bootstrap analysis using phantom variables was performed to verify the significance of the indirect effect. 

In the statistical analysis, statistical significance was determined based on a significance level of 5%. 

## 4. Results

### 4.1. General Characteristics of Participants

The participants consisted of 167 males (49.0%) and 174 females (51.0%), with 71 participants in their 20s (20.8%), 123 in their 30s (36.1%), 83 in their 40s (24.3%), 51 in their 50s (15.0%), and 13 in their 60s or older (3.8%). In terms of religion, there were 73 Presbyterians (21.4%), 30 Catholics (8.8%), 45 Buddhists (13.2%), 4 participants with other religious beliefs (1.2%), and 189 non-religious participants (55.4%). The income level was high for 17 participants (5.0%), medium for 198 participants (58.1%), and low for 126 participants (37.0%). Regarding the use of smartphones and the internet, 339 participants (99.4%) answered “Yes” and two participants (0.6%) answered “No.” In terms of marital status, there were 167 single participants (49.0%), 164 married participants (48.1%), eight divorced participants (2.3%), and two others (0.6%) (Table 1).

### 4.2. Descriptive Statistics and Correlation of Research Variables

The mean and standard deviation were calculated to understand the level of the research variables measured in this study. 

The average level of health beliefs related to the prevention of pandemics was 2.72 points for perceived severity and 2.13 points for perceived disability, both out of 4 points. The average level of attitude toward pandemic prevention was 4.04 points out of 5 points. The average level of the sub-factors was 4.14 points for personal belief attitude, 4.01 points for knowledge attitude, 3.96 points for normative attitude, 3.88 points for emotional attitude, and 3.96 points for intentional attitude. The average level of the subjective norm was 5.77 points out of 7 points, the average level of perceived behavioral control was 2.74 points out of 7 points, and the average level of intention to prevent a pandemic was 5.77 points out of 7 points. The average level of pandemic prevention behavior was 3.94 points out of 5 points, and the average level of the sub-factors was 3.49 points for personal hygiene, 4.41 points for quarantine, and 3.70 points for social distancing. 

In addition, skewness and kurtosis were calculated to determine whether the assumption of normality was satisfied for the variables. An absolute measure of skewness less than 3 and an absolute measure of kurtosis less than 10 indicated that it was close to the normal distribution [41], and all variables were found to satisfy the assumption of normality (Table 2).

As a result of correlation analysis, perceived severity had a statistically significant negative correlation with perceived behavioral control (r = −0.130, *p* < 0.05). Perceived disability had a significant negative correlation with attitudes toward pandemic prevention behavior (r = −0.380, *p* < 0.001) and with the subjective norm (r = −0.300, *p* < 0.001), while having a significant positive correlation with perceived behavioral control (r = 0.358, *p* < 0.001).

There were significant positive (+) correlations between attitudes toward pandemic prevention behavior (r = 0.713, *p* < 0.001), subjective norms (r = 0.692, *p* < 0.001), and pandemic prevention intention. There was a significant negative correlation between perceived behavioral control and pandemic prevention behavioral intentions (r = −0.427, *p* < 0.001).

There was a significant negative correlation between perceived behavioral control and pandemic prevention behavior (r = −0.394, *p* < 0.001), and there was a significant positive (+) correlation between the pandemic prevention intention and the pandemic prevention behavior (r = 0.696, *p* < 0.001) (Table 3). Therefore, hypothesis 2 was supported.

### 4.3. Exploratory Factor Analysis

As a result of the exploratory factor analysis, and as a result of performing Harman’s one factor test, 10 factors were extracted for the variables of this study. The total variance explained by 10 factors was 66.85%, and the largest explanatory power extracted by one factor was 11.55%. Since this represents a numerical value below the reference value, it can be determined that the same method bias did not occur in the measurement model of this study. Another review method involves checking the correlation coefficient between latent variables. According to Pavlou et al. [42], the correlation coefficient between all latent variables should be less than 0.90 in order to avoid the problem of same method bias. The maximum correlation coefficient between the latent variables presented in the correlation analysis is 0.713, so it can be confirmed that it meets the standard value. Therefore, it was judged that the same method bias problem did not occur in this study.

### 4.4. Structural Model Analysis

#### 4.4.1. Validation of the Model

In this study, confirmatory factor analysis was performed to verify the validity of the model, and convergent validity and discriminant validity were used. To test the convergence validity, the conceptual reliability (CR) and the average variance extracted (AVE) for each variable were calculated. Convergence validity was judged to be good when the CR was 0.70 or more and the AVE was 0.50 or more [31]. Since it exceeded the standard in all items, this model was judged to have high convergent validity. To verify discriminant validity, correlation coefficients between latent variables and their 95% confidence intervals were calculated. If the 95% confidence interval of the correlation coefficient did not contain 1 or −1, the discriminant validity was judged to be acceptable [43,44]. Since the confidence interval of the correlation coefficient between all variables did not include −1 or 1, the discriminant validity was judged to be good.

#### 4.4.2. Hypothetical Model Fit Test and Model Analysis

**Figure 2:**
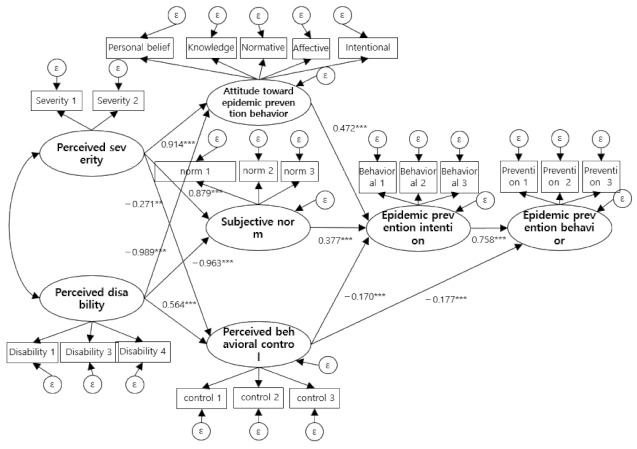
Hypothetical model of research. ** *p* < 0.01 *** *p* < 0.001.

Regarding the fit of the research model, the value of χ^2^ (df = 176) was 437.373 (*p* < 0.001). Since the χ^2^ value is greatly affected by the sample size and model complexity, other goodness-of-fit indices were identified [44]. As a result, RMR was 0.054, GFI was 0.889, and RMSEA was 0.066, showing generally good levels of NFI at 0.898, TLI at 0.923, and CFI at 0.936. The hypothetical model was found to fit the data well. See Figure 2.

#### 4.4.3. Structural Model Parameter Estimation and Effect Analysis

The results of the analysis of the path estimation and significance of the structural model are shown in Table 4, and a total of 11 direct paths were found to be significant. Perceived severity (β = 0.914, *p* < 0.001) and perceived disability (β = −0.989, *p* < 0.001) had a significant effect on attitudes toward pandemic prevention, and the explanatory power was 83.2%. Perceived severity (β = 0.879, *p* < 0.001) and perceived disability (β = −0.963, *p* < 0.001) had a significant effect on the subjective norm, and the explanatory power was 78.1%. Perceived severity (β = −0.271, *p* < 0.01) and perceived disability (β = 0.564, *p* < 0.001) had a significant effect on perceived behavioral control, and the explanatory power was 22.6%. Attitudes toward pandemic prevention behavior (β = 0.472, *p* < 0.001), subjective norms (β = 0.377, *p* < 0.001), and the perceived behavioral control (β = −0.170, *p* < 0.001) had a significant effect on pandemic prevention intentions, and the explanatory power was 78.8%. Pandemic prevention intention (β = 0.758, *p* < 0.001) and perceived behavioral control (β = −0.177, *p* < 0.001) had a significant effect on pandemic prevention behavior, and the explanatory power was 73.7%.

In this study, effect decomposition was performed using the bootstrapping method to identify the structural relationship of the research model and to verify its significance; the significance analysis result of the indirect effect was as follows. Perceived severity (β = 0.808, *p* < 0.01) and perceived disability (β = −0.925, *p* < 0.01) had significant indirect effects on the intention to prevent pandemics. Perceived severity (β = 0.661, *p* < 0.001) and perceived disability (β = −0.801, *p* < 0.01), pandemic prevention attitude (β = 0.358, *p* < 0.05), perceived disability, and perceived behavioral control (β = −0.129, *p* < 0.001) had significant indirect effects on pandemic prevention behavior. The indirect effect of subjective norms on pandemic prevention behavior was not significant. Therefore, in this study, Hypothesis 1 and Hypothesis 3 were supported, but Hypothesis 4 was only partially supported.

## 5. Discussion

In this study, a hypothetical model for pandemic prevention behavior in adults was established by applying the HBM and the TPB, and the suitability of the model was verified. In this regard, it is intended to review the effects of factors related to pandemic prevention behavior in adults, and discuss suggestions for interventions to improve pandemic prevention behaviors in the future. 

First, health beliefs about epidemic prevention behavior were verified in relation to perceived seriousness and disability. The effect of the perceived severity of pandemic prevention behaviors on attitudes toward pandemic prevention behaviors, subjective norms, and perceived behavioral control was examined. Individuals’ perceptions of the severity of the prevention and incidence reduction effects of pandemic prevention behaviors had a direct effect on their attitudes toward pandemic prevention behaviors, subjective norms, and perceived behavioral control. It also had an indirect effect on pandemic prevention intentions and behaviors. In other words, the higher the perceived severity of an pandemic prevention action, the more positively the attitude toward the pandemic prevention behavior changes, thereby creating the environment to implement the pandemic prevention behavior. 

The results of studies on the perceived severity of pandemics are varied. The results of this study supported the existing study in TPB, which stated that attitudes toward behavior, subjective norms, and perceived behavioral control could predict behavioral intentions, enabling a simple and accurate understanding of human behavior [13]. Furthermore, these results are consistent with the results of perceived sensitivity in a study on the factors affecting the prevention of COVID-19 infection among residents of a certain area in South Korea [40]. However, they conflict with the results of perceived severity [45] in a study conducted among residents of northern Iran. Although perceived severity was an important variable for taking preventive measures in general, individuals had a high sense of vulnerability to the disease, and negative emotions were also affected by the attitudes of individuals who regarded the disease as dangerous [2,45]. Therefore, it seems necessary to promote the prevention of pandemics by controlling perceptions of severity in relation to individual attitudes and subjective norms. 

Second, we investigated the effect of perceived disability on pandemic disease prevention behavior, attitudes toward pandemic prevention behavior, subjective norms, and perceived behavioral control. Perceived disability had a direct effect on attitudes toward preventive behavior, subjective norms, and perceived behavioral control, and showed an indirect effect on the intentions and performance of preventive behavior. This is consistent with the results of several studies showing that, unlike perceived severity, perceived disability interfered with prevention behaviors [28,40,45]. The structural model path of this study revealed that the positive attitude toward prevention behavior was reduced when more obstacles were perceived. Moreover, where participants also perceived less pressure from people around them to always follow the precautionary guidelines, the difficulty they felt about always following the precautionary guidelines increased. These results also seem consistent with research results that show perceived disability to be indirectly related to behavioral performance through behavioral intentions [46,47].

In addition, there was a positive correlation between epidemic prevention attitudes, subjective norms, and perceptual behavior control among adults. This result is the same as the previous verification studies on the TPB [14,20].

Third, we examined the effects of attitudes, subjective norms, and perceived behavioral control toward pandemic prevention behaviors on pandemic prevention behavior intentions. The explanatory power of attitudes, subjective norms, and perceived behavioral control toward pandemic prevention behavior on performance intention was reported as 78.8%. It was found that attitude, subjective norms, and perceived behavioral control, which were variables of the planned behavior theory, had a direct effect on the intention to implement pandemic prevention behavior, with perceived severity and disability having an indirect effect. These results supported previous study results [15,18,47], indicating that attitudes and perceived behavioral control predict performance intentions by applying TPB-based educational interventions. In this study, the subjective norm, which referred to social pressures that affect the performance of behaviors, had a direct effect on the intention of prevention behavior. Some studies have verified that subjective norms have a direct effect on the performance intentions of behaviors [26,46], which seems to require follow-up studies. The effects of education on attitudes, norms, and behavioral control for behavior promotion [14,15,17,24] have been proven in several studies. Pandemic prevention education programs that focus on factors that promote planned behaviors provide the information required to make decisions to improve pandemic prevention behaviors, thereby reducing the spread and transmission rate of pandemics.

Fourth, the effect of perceived behavioral control and the intention to prevent a pandemic on pandemic prevention behavior was examined. The explanatory power of perceived behavioral control and implementation intention on preventive behavior was reported as 73.7%. Perceived severity, disability, and attitudes toward pandemic prevention behavior had an indirect effect, and the subjective norm was not a predictor variable. Perceived behavioral control was a variable for which both direct and indirect effects were predicted. Perceived behavioral control was a determinant of behavioral transition and behavioral change, and was influenced by education [30,32].

From the respondents’ point of view, detailed education on pandemic prevention guidelines not only influenced their confidence in solving problems related to practicing preventive actions, but also induced effective preventive actions as a result.

The study result [46], which revealed that social control and education on pandemic prevention guidelines were essential for increasing preventive behavior, suggests that education and social control regarding detailed practical behaviors play an important role in the performance of pandemic prevention behaviors. In other words, from the point of view of the government regulatory body, in order to prevent an epidemic, it is necessary to exercise social control with a certain degree of compulsion to control prevention and promotion activities. It can be surmised that healthcare providers should provide training on detailed practical actions under these controls.

## 6. Conclusions

This study attempted to identify factors that affected the pandemic prevention behavior in adults, and to suggest interventions to promote pandemic prevention behavior in the future. The structural model of this study was found to be suitable for predicting pandemic prevention behavior, which was significant because the model was developed based on the HBM and the TPB. The factors affecting pandemic prevention behavior were the intention of the pandemic prevention behavior, and the perceived behavioral control of the pandemic prevention behavior. Attitudes, subjective norms, and perceived behavioral control in relation to pandemic prevention behaviors directly affected respondents’ intention to implement pandemic prevention behaviors. Attitude, subjective norms, and perceived behavioral control of pressure on prevention behaviors had an effect on attitude, subjective norms, and perceived behavioral control. Therefore, to promote the prevention of pandemics, it is necessary to increase the severity of the prevention of pandemics and improve awareness of pandemics at the national level. The development of a pandemic prevention education program will be required, in order to increase positive attitudes toward pandemic prevention behavior and to raise subjective norms, as will institutional and environmental campaigns and awareness to reduce factors that hinder prevention behaviors.

This study has the following limitations. First, perceived severity and disability, which are part of the HBM, were measured as individual health beliefs that affected the performance of pandemic prevention behavior. Second, other factors that could affect the practice of prevention behavior were not included in this study. Furthermore, it cannot be forgotten that pandemic transmission cannot be 100% prevented by performing preventive behaviors, due to the existence of other influencing factors, such as the development of therapeutic agents and vaccines, and the emergence of new variants. Perceived severity and disability were measured as individual health beliefs; future studies should integrate institutional and environmental variables.

Based on the results, we suggest the following actions. First, further research is required to identify more variables, in addition to those addressed in the HBM and the TPB, that can be analyzed in relation to adults’ health behavior during pandemics. Second, the development of campaigns and educational programs is required to bring about effective social change in attitudes, subjective norms, and perceived behavioral control, all of which are factors that have a significant influence on the intention to perform pandemic prevention behaviors, practice preventive actions, and manage those infected by the pandemic. Third, while this study identified levels of pandemic prevention behavior through self-reported questionnaires, observational studies that can measure the direct effect of behavioral practices on prevention are needed.

## Figures and Tables

**Table 1:** General characteristics of the study subjects.

Item	Division	N	%
Gender	Male	167	49
	Female	174	51
Age	20s	71	20.8
	30s	123	36.1
	40s	83	24.3
	50s	51	15
	60s or older	13	3.8
Religion	Presbyterian	73	21.4
	Catholic	30	8.8
	Buddhist	45	13.2
	Other	4	1.2
	Non-religious	189	55.4
Income level	High	17	5
	Medium	198	58.1
	Low	126	37
Smartphone and internet use	Yes	339	99.4
	No	2	0.6
Marital status	Single	167	49
	Married	164	48.1
	Divorced	8	2.3
	Other	2	0.6
Total		341	100

**Table 2:** Descriptive statistics of the study variables.

Variable		Minimum	Maximum	Mean	Standard Deviation	Skewness	Kurtosis
Health beliefs on pandemic prevention behavior	Perceived severity	1	4	2.72	0.67	−0.28	0.05
	Perceived disability	1	4	2.13	0.6	0.25	0
Attitude toward pandemic prevention behavior	Personal belief attitude	1	5	4.14	0.78	−1.7	3.47
	Knowledge attitude	1	5	4.01	0.74	−1.15	2.3
	Normative attitude	1	5	3.96	0.78	−0.68	0.38
	Affective attitude	1	5	3.88	0.71	−0.91	1.54
	Intentional attitude	1	5	3.96	0.8	−0.81	0.88
	Total	1.24	5	4.04	0.61	−1.52	3.44
Subjective norm		1.5	7	5.77	1	−1.39	2.87
Perceived behavioral control		1	6.67	2.74	1.37	0.83	−0.18
Pandemic prevention intention		1.2	7	5.77	0.96	−1.42	3.09
Pandemic prevention behavior	Personal hygiene rules	1	5	3.49	0.76	−0.39	0.1
	Quarantine rules	1.33	5	4.41	0.65	−1.32	1.55
	Distancing	1.25	5	3.7	0.71	−0.19	−0.26
	Total	1.43	5	3.94	0.54	−0.63	1.06

**Table 3:** Correlation of study variables.

	Perceived Severity	Perceived Disability	Pandemic Prevention Attitude	Subjective Norm	Perceived Behavioral Control	Pandemic Prevention Intention	Pandemic Prevention Behavior
Perceived severity	1						
Perceived disability	0.158 **	1					
Attitude toward pandemic prevention behavior	0.061	−0.380 ***	1				
Subjective norm	0.096	−0.300 ***	0.667 ***	1			
Perceived behavioral control	−0.130 *	0.358 ***	−0.341 ***	−0.294 ***	1		
Pandemic prevention intention	0.097	−0.346 ***	0.713 ***	0.692 ***	−0.427 ***	1	
Pandemic prevention behavior	0.130 *	−0.309 ***	0.556 ***	0.522 ***	−0.394***	0.696 ***	1

* *p* < 0.05 ** *p* < 0.01 *** *p* < 0.001.

**Table 4:** Parameter estimation and effect analysis of the structural model.

Path	Direct Effect			Indirect Effect	Effect	SMC
	β	C.R.	*p*	β(*p*)	β(*p*)	
Perceived severity → Pandemic prevention attitude	0.914	5.009 ***	<0.001		0.914 (<0.001)	0.832
Perceived disability → Pandemic prevention attitude	−0.989	−6.414 ***	<0.001		−0.989 (<0.001)	
Perceived severity → Subjective norm	0.879	5.283 ***	<0.001		0.879 (<0.001)	0.781
Perceived disability → Subjective norm	−0.963	−6.847 ***	<0.001		−0.963 (<0.001)	
Perceived severity → Perceived behavioral control	−0.271	−3.004 **	0.003		−0.271 (0.003)	0.226
Perceived disability → Perceived behavioral control	0.564	6.295 ***	<0.001		0.564 (<0.001)	
Perceived severity → Pandemic prevention intention				0.808 (0.002)	0.808 (0.002)	0.788
Perceived disability → Pandemic prevention intention				−0.925 (0.003)	−0.925 (0.003)	
Pandemic prevention attitude → Pandemic prevention intention	0.472	5.250 ***	<0.001		0.472 (<0.001)	
Subjective norm → Pandemic prevention intention	0.377	4.186 ***	<0.001		0.377 (<0.001)	
Perceived behavioral control → Pandemic prevention intention	−0.17	−4.235 ***	<0.001		−0.170 (<0.001)	
Perceived severity → Pandemic prevention behavior				0.661 (<0.001)	0.661 (<0.001)	0.737
Perceived disability → Pandemic prevention behavior				−0.801 (0.002)	−0.801 (0.002)	
Pandemic prevention attitude → Pandemic prevention behavior				0.358 (0.026)	0.358 (0.026)	
Subjective norm → Pandemic prevention behavior				0.286 (0.085)	0.286 (0.085)	
Pandemic prevention intention → Pandemic prevention behavior	0.758	11.215 ***	<0.001		0.758 (<0.001)	
Perceived behavioral control → Pandemic prevention behavior	−0.177	−3.583 ***	<0.001	−0.129 (<0.001)	−0.306 (<0.001)	

** *p* < 0.01 *** *p* < 0.001.

## Data Availability

No new data were created or analyzed in this study. Data sharing is not applicable to this article.
